# Community-based group rehabilitation program for stroke patients with dysphagia on quality of life, depression symptoms, and swallowing function: a randomized controlled trial

**DOI:** 10.1186/s12877-023-04555-0

**Published:** 2023-12-20

**Authors:** Chen Yang, Fei Zhao, Chunqing Xie, Yaowen Zhang, Zulin Dou, Xiaomei Wei

**Affiliations:** Department of Rehabilitation Medicine, The Third Affiliated Hospital, Sun Yat-sen University, Guangzhou, China

**Keywords:** Stroke, Dysphagia, Deglutition, Group rehabilitation, Swallowing function, Depression, Quality of life

## Abstract

**Background:**

Community-based exercise programs have demonstrated potential for implementation in older adults; however, it remains imperative to ascertain whether this strategy will yield comparable benefit in stroke patients with dysphagia.

**Methods:**

This was a single blinded, randomized, matched pairs clinical trial. Sixty-four stroke patients with dysphagia were recruited from patients who had been discharged the Rehabilitation Department of the Third Affiliated Hospital of Sun Yat-sen University. A single blinded, randomized and controlled trial was conducted. Participants were randomly assigned to either the intervention group (*n* = 32) or the control group (*n* = 32). Patients in the intervention group received health education followed by swallowing function training in community public spaces for 5 days every week over an eight-week period (60 minutes per day). Patients in the control group received swallowing rehabilitation training, and booster educational information about dysphagia, as well as instructions on how to improve quality of life. Swallowing function (Functional Oral Intake Scale (FOIS) and Standardized Swallowing Assessment (SSA)), depressive symptoms (Geriatric Depression Scale-15), and quality of life (Swallowing-Quality of Life, SWAL-QOL) were assessed before and after all the treatment.

**Results:**

Before treatment, the two groups did not differ statistically. After the intervention, the swallowing function (SSA and FOIS) showed a significant improvement in both groups (All *p* < 0.001). But there was no significant difference in Functional Oral Intake Scale change between groups (*P* = 0.479). Compared with the control group, the intervention group had a significant improvement in depressive symptoms (*P* = 0.002), with a greater reduction in the number of depressed patients (13 to 6).The control group showed no significant improvements in depressive symptoms or a reduction in the number of depressed patients before and after treatment (*P* = 0.265, 14 to 12). The Swallowing-Quality of Life scores showed significant improvement in both the intervention and control group (*P* < 0.001). Specifically within Swallowing-Quality of Life sub-domains, greater changes were observed in symptoms and frequency (*P* < 0.001), communication (*P* = 0.012), and sleep (*P* = 0.006) for participants in the intervention group. And the cost-effectiveness of group rehabilitation surpasses that of rehabilitation training.

**Conclusion:**

Community-based group rehabilitation program is more effective than traditional treatment in improving patients’ depressive symptoms and quality of life, as well as being more cost-effective.

## Introduction

Stroke is complicated by oropharyngeal dysphagia in 29 to 81% of patients [1, 2]. Up to 40% of these individuals continue to experience swallowing difficulty even after a year later [3], which is associated with an increased risk of consequences such as aspiration pneumonia [4], dehydration, and malnutrition [5]. In cases patients are unable to safely consume food or drink orally, this series of complications may decrease patient’s quality of life [6]. Furthermore, non-oral or limited oral feeding and the risk of aspiration restrict patients’ social activities [7], resulting in social isolation that worsens depression [8].

The common outcome for patients with post-stroke dysphagia is that those whose function does not improve choose to transfer to another hospital for further treatment [9–11], while those whose function improves eventually return to their families or communities. For patients returning to the family and community, the most common late treatment methods at home and abroad include remote follow-up guidance by nurses [12] or on-site service provided by SLP [13]. However, these methods are not suitable for general promotion due to poor compliance or high requirements in terms of time and cost-effectiveness. At present, most of the community-based swallowing rehabilitation programs are conducted for healthy elderly individuals with chronic diseases, and there is a lack of group training specifically designed for patients with dysphagia [14]. Other studies have shown that when patients engage in therapeutic activities in a group social environment, not only can they improve physical function, but they may reach a larger number of people, improve interpersonal relationships and adherence to guidelines, and provide reduction in health costs [15]. Furthermore, the inclusion of leisure activities also contributes to preventing health decline and social isolation in stroke patients [16].

Group rehabilitation programs have become increasingly popular in recent years. Studies had demonstrated the potential of a community-based exercise programs in older adult [17]. However, there are few reports on the efficacy of group rehabilitation programs for dysphagia. Given the diversification of dysphagia problems in patients, and the current resource constraints faced by discharged individuals impede the advancement rehabilitation by hindering their access to hospital resources. Based on the effectiveness and shortcomings described the previous literature, this study designed a simple community-based group rehabilitation program to evaluate the effect of the program on quality of life, depressive symptoms, and swallowing function in patients with dysphagia. Moreover, the clinical and cost-effectiveness of a community-based group rehabilitation program for stroke patients with dysphagia was assessed to determine its feasibility and acceptability.

## Methods

### Study design

The study design was an investigator-initiated, prospective, single-blinded, randomized controlled trial. Due to the particularity of intervention measures, it was not feasible to blind the intervener, therefore, only the evaluator remained blinded. The study was registered at Chictr.org in February 2022. (Chinese Clinical Trial Registry Unique Identifier ChiCTR2200056768, registration time 15/02/2022), and received approval from the Clinical Medical Research Ethics Committee of The Third Affiliated Hospital of Sun Yat-Sen University. The study began in January 2021 and concluded in January 2022.

### Randomization

A computer-based random number sequence was generated by a SLP who was not involved in eligibility assessment, data collection, or analysis. The investigators were kept unaware of the allocations through the use of sequentially numbered opaque, sealed envelopes for participant. Participants were randomly assigned in a 1:1 ratio to either the intervention group or the control group. Due to the inherent nature of the interventions, blinding of the staff responsible for their administration was not feasible.

### Participants

A total of 120 stroke patients were recruited from patients who had been discharged the Rehabilitation Department of The Third Affiliated Hospital of Sun Yat-sen University. Two SLPs utilized a standard toolkit for swallowing function assessment on the enrolled patients. Fifty-two participants did not meet the inclusion criteria and were excluded, while the remaining four refused to participate. The remaining 64 eligible patients were informed about the trial and was randomized into two groups (1:1).

### Eligibility criteria

*Inclusion criteria:* (a) Diagnosis of a cerebral hemorrhage or cerebral infarction according to World Health Organization (WHO)'s definition of stroke. Besides, the patients had the first episode of the disease, aged 60 years or older; (b) Mini-Mental State Examination score was⩾22; (c) Dysphagia was identified by SLPs through the bedside Water Swallowing Test, as measured by levels II (t > 5 s), III, IV, and V; and dragonized through either Videofluoroscopic swallowing study or Flexible endoscopic evaluation of swallowing; (d) could follow the brief rehabilitation and evaluation instructions. All the participants or their families gave informed written consent to the study.

*Exclusion criteria:* (a) Patients struggled with instructions or who were unable to complete the entire rehabilitation program; (b) had history of diseases affecting swallowing function (such as Parkinson’s disease or motor neuron disease); (c) history of swallowing treatments or history of radiotherapy and chemotherapy in head and neck.

*Drop-off criteria:* (a) Participants with poor treatment compliance; (b) be absent with two consecutive or three non-consecutively training sessions.

### Interventions

To ensure compliance, a 60-minute self-management program conducted by SLPs prior to randomization for all participants. This program included explanation of the role of SLPs in treatment sessions and recommendations for feeding aids with compensations/adjustments.

#### Intervention group

The group rehabilitation program comprised daily 60-minute sessions, five times per week for a duration of 8 weeks. No more than five participants and two SLPs were assigned to each team. The SLPs have over 5 years of professional practice experience. Additionally, family members or other caregivers were permitted to be an observer during the sessions. The group rehabilitation program included:

1. Rehabilitation oral and facial exercises: including facial exercises, lip exercises, tongue exercises, jaw exercises, respiration muscle exercises, Masako exercises, Shaker exercises, Airway Protection Techniques.
2. Game-based surface electromyographic biofeedback training (GBsEMGBF): The training protocol utilizes a XY¯K¯TY¯I type swallowing nerve and muscle electrical stimulator (Made in China), integrated with game-based surface electromyographic biofeedback. All operations used disposable adhesive electrodes, with the two main electrodes affixed to the hyoid bone and the mandibular joint respectively. The reference electrode was affixed to the 2 cm side of the main electrode. Before starting training, each patient received necessary explanations, and Mendelssohn’s gimmick was informed of the standard waveform when he swallowed. The instruent was set to” GAME mode” during training sessions. The patients were instructed to follow Mendelssohn’s swallowing techniques, and we observed whether the waveform on the screen was consistent with the standard waveform. We ensured that both waveforms matched in order to achieve a higher score. The patients earned the corresponding achievements and rewards through accumulation of scores.
3. Participants experience sharing: Select patients who exhibit discernible treatment outcomes to effectively communicate their treatment experiences, thereby enhancing patients’ confidence in the efficacy of the treatment.
4. Individual direct feeding training, including adjustments to bolus volume, food texture, and posture compensation techniques, lasts for approximately 20 minutes. All of the basic and specialized training can be timely adjusted based on evaluations by the medical team and modified by the SLP according to each patient’s specific condition.

Each session lasted 40 min of basic training, and the total duration was 1.5 h. The patients were allowed to take a 1-min break between each basic training session.

This study used a check-in and punch-in system to guarantee that patients completed the required amount of instruction. Additionally, our program design allowed for flexibility; if a participant missed a training session, we offered make-up sessions. Strategies for increasing training responses include: (a) elucidating the potential benefits of the training procedure in aiding patients’ recover from dysphagia; (b) providing prizes to participants before the completion of the training; and (c) guaranteeing to keep participants’ personal information confidential.

#### Control group

Participants underwent the same rehabilitation oral and facial exercises as the Intervention group, and received booster educational information on dysphagia management and strategies to enhance quality of life through handbooks. The handbooks had been recorded by professional SLPs for the visualization, specificity, and simplification of patient training content. The handbooks contained the same essential information given to intervention group during the corresponding period.

### Outcome assessment tools

All the participants were assessed immediately before and after the 8-week training period.

#### Evaluation of swallowing function

(1) Standardized Swallowing Assessment (SSA). The scale consists of three parts: clinical examination, a 5 mL water swallowing test, and a 60 mL water swallowing test. The score ranges from 18 to 46 points. The higher the score, the worse the swallowing function [18]. (2) Functional Oral Intake Scale (FOIS). We used the Functional Oral Intake Scale to evaluate the ability of oral feeding of the patients. A higher score indicates a better oral feeding ability [19].

#### Evaluation of ill-emotion

15-item Geriatric Depression Scale (GDS-15). 15-item Geriatric Depression Scale was used to evaluate depressive symptoms in elderly population. Participants responded with either “yes” or “no” of the 15 items. A “yes” was assigned one point, while a “no” was assigned a score of zero. A score of 5 or more points diagnoses depression [20].

#### Evaluation of quality of life

Swallowing-related Quality of Life (SWAL-QOL). We used Swallowing-related Quality of Life to evaluate the quality of life. The scale consists of 44 items across 11 dimensions (Social, Sleep, Fatigue, Mental health, Communication, Burden, Eating duration, etc.), the total score is 220 points. The higher the score, the better the patient’s quality of life [21].

### Cost-effectiveness analysis

We conducted a cost-benefit analysis from a societal perspective to determine the costs required for group rehabilitation training compared to traditional rehabilitation. We estimated the value of the following resources: medical services, transportation to and from health care facilities, time spent by family and friends in caring for the patient, and time spent by the patient in receiving treatment and so on. And questionnaire surveys and telephone follow-up were conducted to assess the cost effectiveness.

(a) Questionnaire survey: We designed a questionnaire containing questions related to the objectives of the study, including participants’ personal information, study involvement duration and cost, as well as their perceptions and experiences with the research. The questionnaire was distributed to participants either online or in paper format for convenient completion, and then returned to us. (b) Telephone follow-up: The participants were interviewed via telephone and asked a series of questions related to the study. Their responses were carefully recorded in order to gain deeper insights into their perceptions and experiences regarding the research. The outcome of the cost-effectiveness analysis is represented by the incremental cost-effective ratio (ICER), That is, the ratio of the difference between the relative costs and outputs of an intervention and a control. The ICER represents the cost of the intervention required, on average, to achieve an incremental unit of effect compared to the control strategy.

#### Costs

(1) Medical Costs. Monthly costs for medical care were derived from Medical billing and logging and pharmacy costs. Information regarding Medicare enumerate the received medical services along with their corresponding expenses, encompassing both reimbursements made by Medicare and out-of-pocket payments borne by the patient. Pharmacy costs were mainly for the use of thickeners in patients with dysphagia. The frequency of thickener use was calculated based on a daily requirement of 9 g. After checking the pharmacy and consulting the drug that the thickening agent is priced at 10 yuan per package, which contains 3 g each. By multiplying the daily dose by the unit price, an estimate of the total cost during hospital stay can be obtained. (2) Nonmedical Costs. Nonmedical costs were estimated on the basis of costs associated with the time spent by caregivers, the time spent by the patient, and trip cost, Volunteer-related costs are also included. The cost of the time patients spent receiving treatment was estimated on the basis of the 1.5 hours spent per day receiving treatment in the hospital or community. The number of caregiver staff hours worked per day was estimated by multiplying the number of hours spent by patients by 1.2. Travel costs were estimated on the basis of the number of miles traveled from the patient’s home to community rehabilitation centers. The cost of volunteer labor was calculated at 20yuan per person per hour for the services provided by treatment volunteers to the patients. The cost of volunteer time was calculated based on the treatment time, which amounted to 3 hours per day. The cost of training and materials encompassed expenses associated with providing rehabilitation treatment-related training to volunteers, including the procurement of instructional resources, remuneration for trainers, and acquisition of low-value consumables. Administrative costs include expenses related to the recruitment, training, scheduling, supervision, and evaluation of volunteers.

### Sample size

We used G*Power (Windows Version 3.1.9.2) to calculate the required sample size, assuming alpha = 0.05, power = 0.80, and effect size(d) = 0.80 with two tails; based on these assumptions, determined to be 52 participants. To account for a conservative dropout rate (10%), at least 57 participants were needed.

### Statistical analysis

All statistical analyses were completed using SPSS version 26.0 (IBM Corp, Armonk, NY, USA), and the significance level was set at *P* < 0.05. Categorical variables were presented as percentage frequencies, whereas descriptive statistics were calculated as a mean (with standard deviation). The chi-square or Fisher’s exact test for categorical data, student’s t-test and Mann-Whitney U test were used to test the significant differences in gains between the two groups. Regarded the changes in the number of depressive symptoms before and after the intervention, the Pearson chi-square test was used for intergroup comparisons, while McNemar’s test was used for intragroup comparisons. Qualitative data on patient reported outcomes and on treatment tolerability are reported. And the Cost-Effectiveness of this study is analyzed.

## Results

**Fig. 1:**
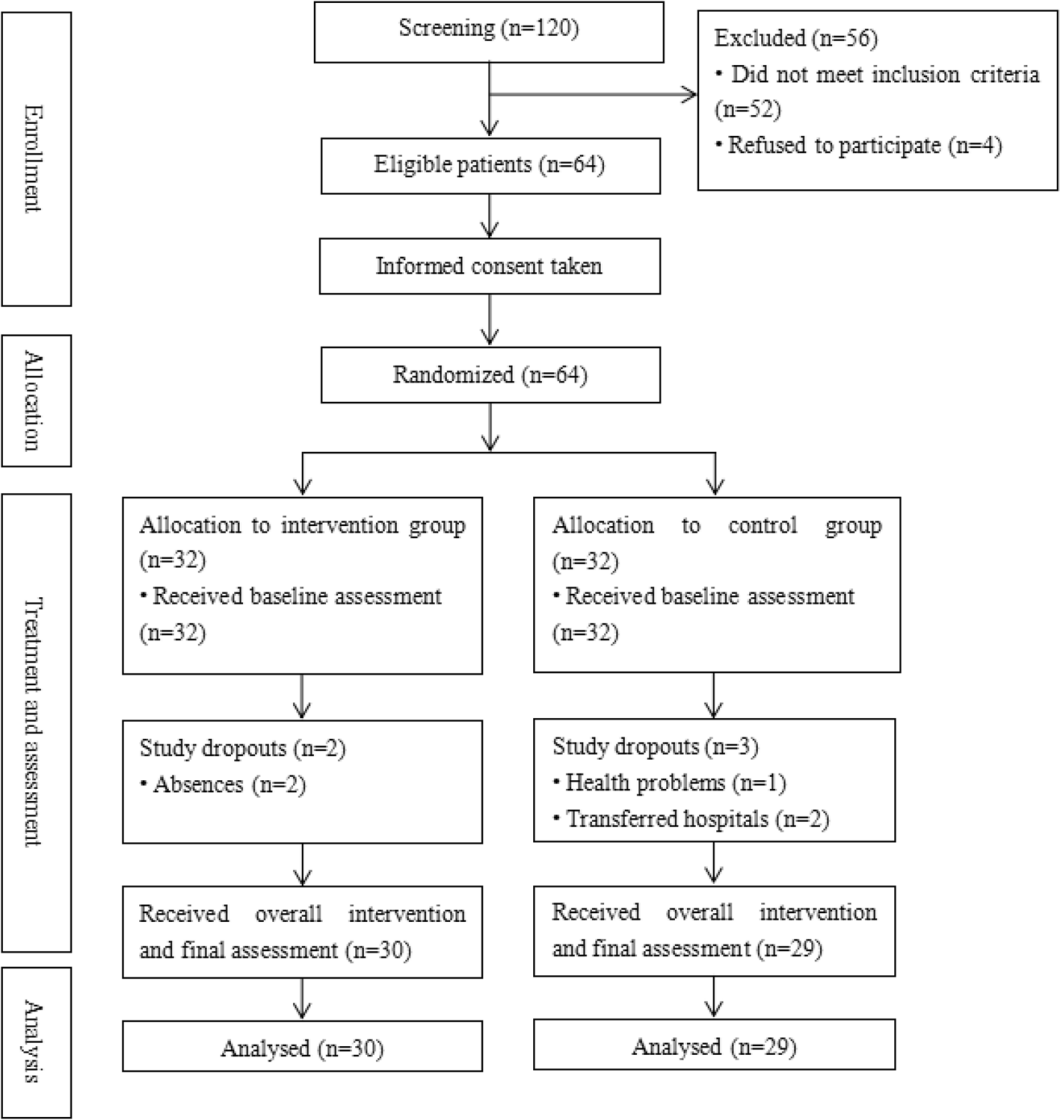
Flowchart of study participants

The flow chart in Fig. 1 illustrates the distribution of participants at each stage of the study. A total of 120 patients were assessed for eligibility, with 52 failing to meet inclusion requirements and four refusing to participate. Of the remaining 64 were randomly assigned, only 59 were finally studied. Two patients in the intervention group were unable to complete all of the group rehabilitation training due to personal reasons. Three control group participants were excluded, one patient was referred for treatment due to disease progression, and two patients were transferred.

### Basic characteristics

Table 1 outlines the sociodemographic and swallowing function assessment features of the patients according to age, sex, time since stroke, stroke type, education level, career, marital status, and the Water swallow test. There were no missing values. In general, the patients of both groups were homogeneous in these characteristics.

**Table 1:** Demographic and clinical characteristics of all patients

Variables	Group		*P*-Value
	Intervention (*n* = 30)	Control (*n* = 29)	
Age (years)			
Mean ± SD(range)	63.200 ± 6.178(56–77)	62.483 ± 6.069(56–80)	0.655
Gender (n, %)			
Male	20 (66.7%)	20 (69%)	0.860
Female	10 (33.3%)	9 (31%)	
Course of disease (months)	2.62 ± 1.298	2.88 ± 1.23	0.428
Stroke type (n, %)			
Infarction	21 (70%)	18 (62%)	0.520
Hemorrhage	9 (30%)	11 (38%)	
Education level (n, %)			
Graduation below primary school	4	3	0.515
Junior high school	12	7	
High school graduation or above	10	15	
College	4	4	
Profession (n, %)			
Did not work	6	5	0.924
Temporary work	11	10	
Steady work	13	14	
Marital status (n, %)			
Married	25	27	0.587
Unmarried	1	1	
Divorced	4	1	
Water swallow test	3.93 ± 0.828	3.90 ± 0.860	0.868

Data are mean (SD) or number

**Fig. 2:**
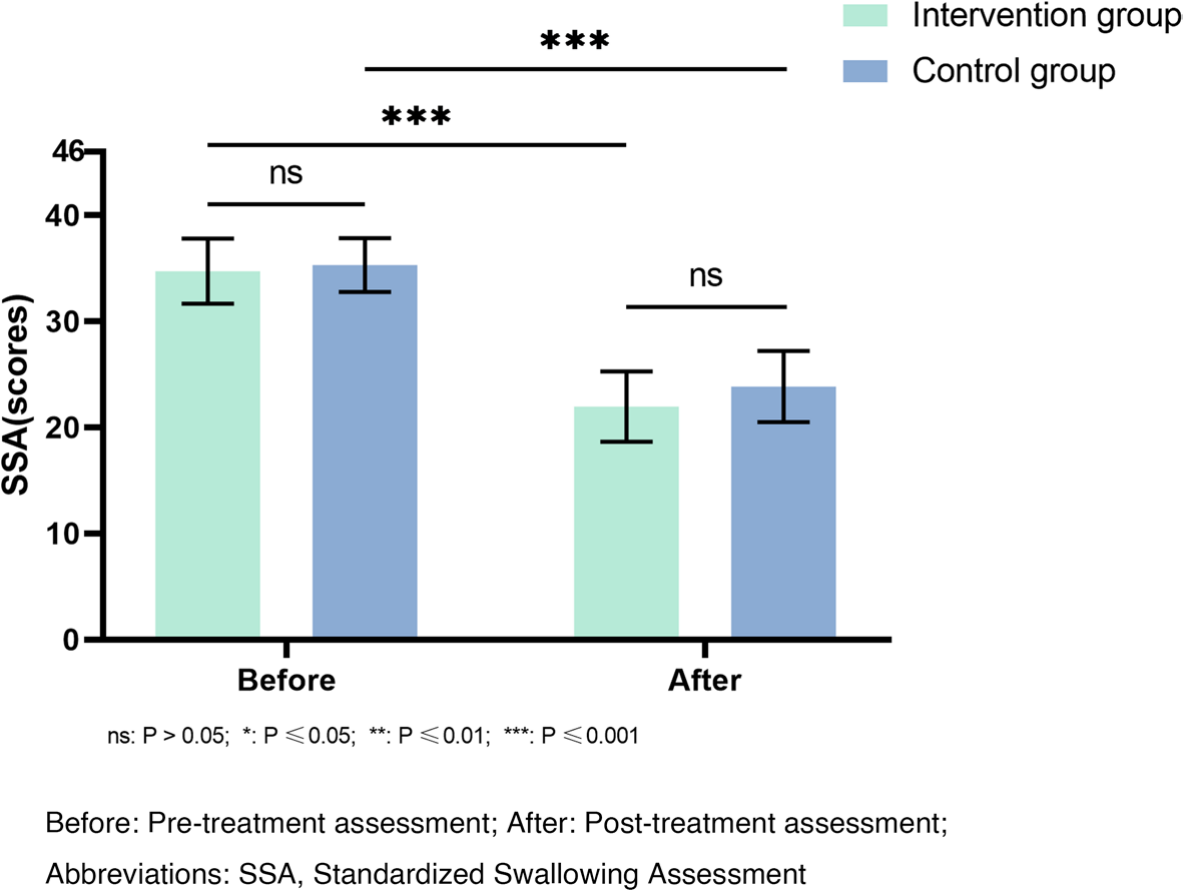
Comparison of the SSA assessment result before and after treatment

**Fig. 3:**
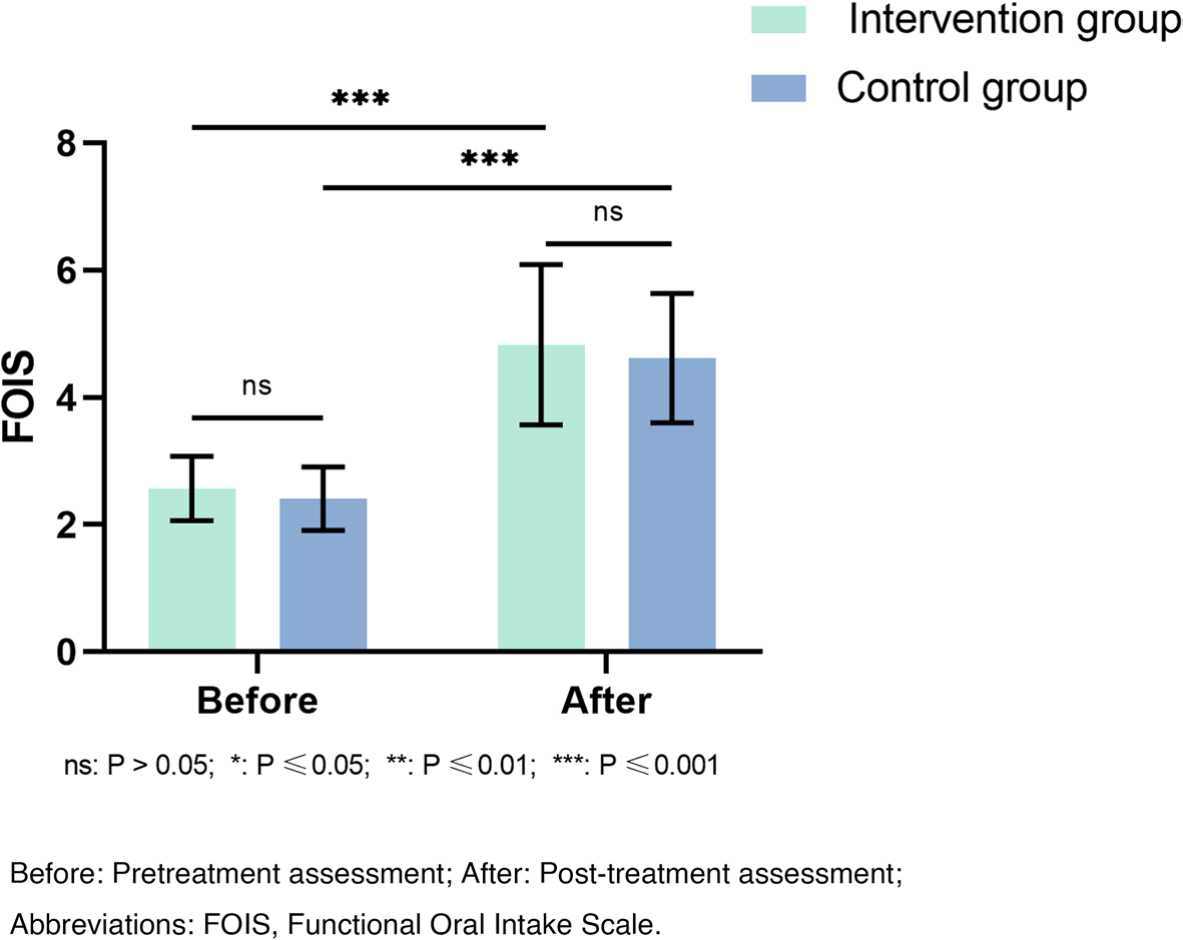
Comparison of the FOIS assessment result before and after treatment

After treatment, both groups showed significant changes in Standardized Swallowing Assessment and Functional Oral Intake Scale compared to before treatment (*P* < 0.001). The intervention group had significantly change scores in Standardized Swallowing Assessment compared to the control group, indicating a significant difference between groups (*P* = 0.033; Fig. 2). However, there was no significant difference in Functional Oral Intake Scale change between the groups (*P* = 0.479; Fig. 3).

**Fig. 4:**
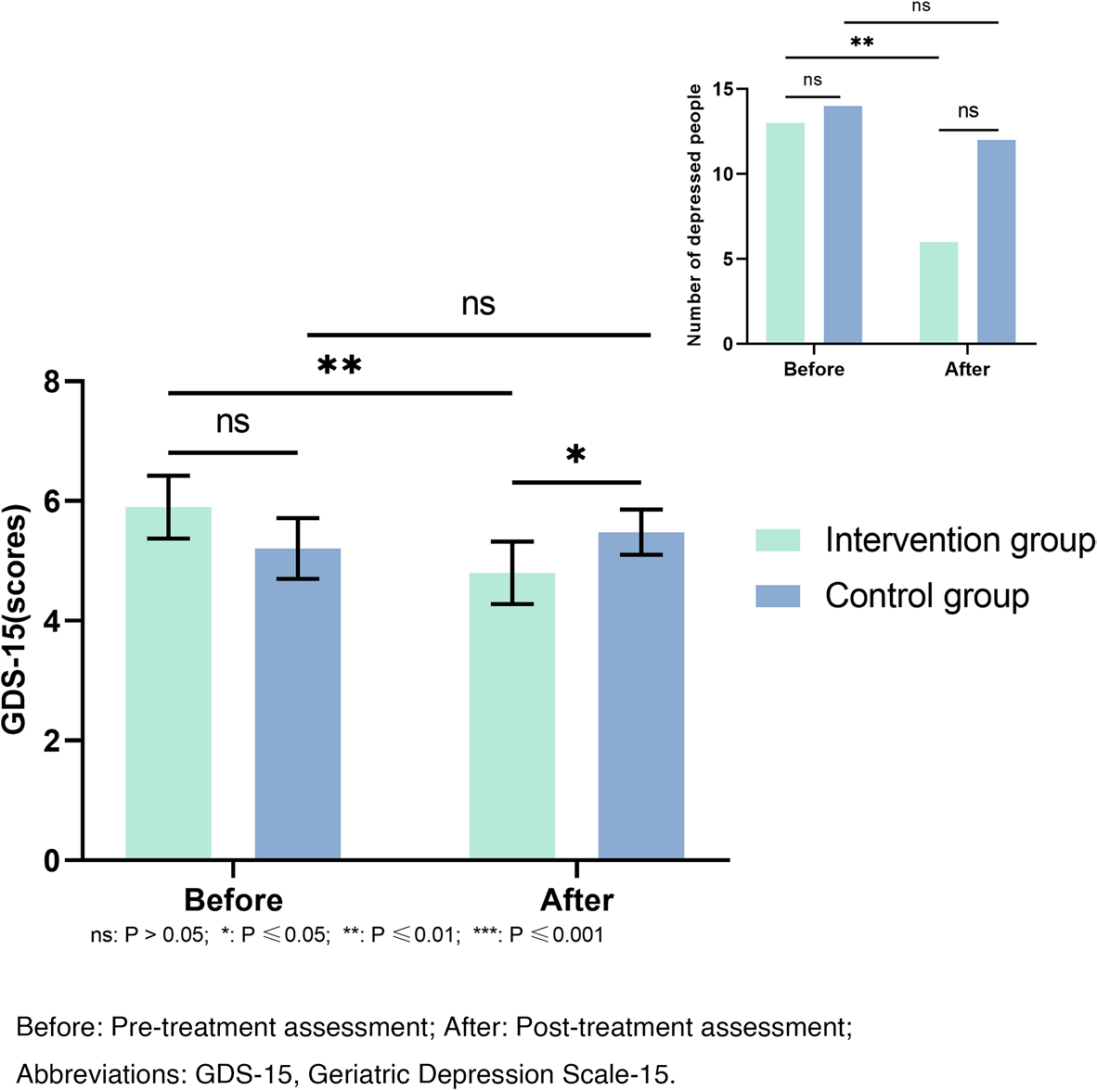
Comparison of the GDS-15 assessment result before and after treatment

We analyzed 15-item Geriatric Depression Scale from two aspects (Fig. 4). That is, the mean and standard deviation and the percentage of patients with depressive symptoms (15-item Geriatric Depression Scale≥5) before and after the intervention. Compared to pre-treatment levels, the 15-item Geriatric Depression Scale score of the control group slightly increased, indicating a worsening tendency in depressive symptoms among these patients. However, there was no significant intra-group difference (*P* = 0.265). The depressive symptoms in the intervention group were significantly improved (*P* = 0.002), and there was a significant difference between groups (*P* = 0.003). The rate of depressive symptoms in the intervention group decreased significantly after treatment (*P* = 0.002). However, the decrease in the control group was not statistically significant (*P* = 0.625).

**Fig. 5:**
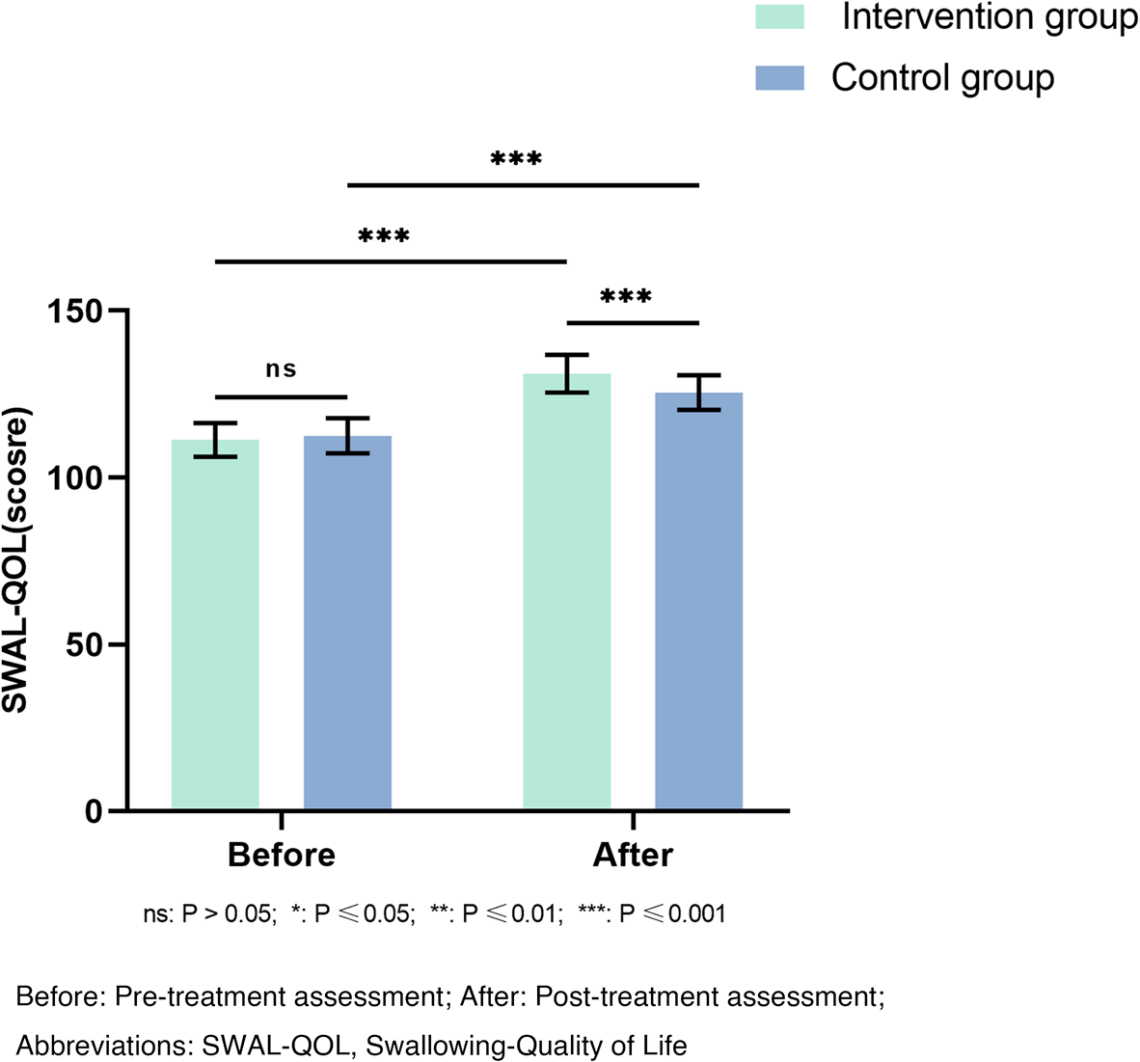
Comparison of the SWAL-QOL assessment result before and after treatment

Swallowing-Quality of Life scores showed significant changes in both groups (*P* < 0.001; Fig. 5) compared to before treatment. The intervention group exhibited significantly greater improvement compared to the control group, with a noticeable difference in the quality of life between two groups (*P* < 0.001). In the Swallowing-Quality of Life sub-domain, the intervention group demonstrated more improvements in symptoms and frequency, communication, and sleep when compared to the control group (Table 2).

**Table 2:** Changes in SWAL-QOL and sub-domain of SWAL-QOL of the two groups between baseline and eight weeks

SWAL-QOL	Test	Group		P-Value
		Intervention (n = 30)	Control (n = 29)	
Total score	Pre Post	111.27 ± 5.010 131.37 ± 5.518	112.48 ± 5.269 125.48 ± 5.255	P1 = 0.367 P2 < 0.001
Burden	Pre Post	5.13 ± 1.833 6.17 ± 1.840	5.41 ± 2.009 5.69 ± 1.854	P1 = 0.577 P2 = 0.325
Fear	Pre Post	9.07 ± 1.015 10.80 ± 1.518	9.10 ± 1.718 10.66 ± 1.317	P1 = 0.921 P2 = 0.697
Eating duration	Pre Post	5.13 ± 1.776 6.40 ± 1.673	5.48 ± 1.825 6.21 ± 1.590	P1 = 0.459 P2 = 0.651
Eating desire	Pre Post	10.70 ± 1.512 11.13 ± 1.358	10.41 ± 1.350 10.69 ± 1.466	P1 = 0.447 P2 = 0.233
Symptoms and frequency	Pre Post	30.23 ± 1.455 36.33 ± 2.496	30.45 ± 2.384 33.48 ± 1.975	P1 = 0.679 P2 < 0.001
Food selection	Pre Post	7.63 ± 1.245 8.73 ± 0.740	7.86 ± 1.407 8.48 ± 0.688	P1 = 0.511 P2 = 0.183
Communication	Pre Post	7.90 ± 1.062 9.27 ± 0.640	8.03 ± 1.180 8.83 ± 0.658	P1 = 0.647 P2 = 0.012
Social	Pre Post	10.53 ± 1.456 12.80 ± 1.827	10.55 ± 1.378 11.83 ± 1.227	P1 = 0.960 P2 = 0.020
Fatigue	Pre Post	8.33 ± 1.124 9.77 ± 1.104	8.45 ± 1.088 9.69 ± 1.105	P1 = 0.692 P2 = 0.790
Sleep	Pre Post	6.53 ± 1.502 7.93 ± 1.172	6.14 ± 0.789 7.17 ± 0.848	P1 = 0.210 P2 = 0.006
Mental health	Pre Post	10.07 ± 2.132 12.03 ± 2.236	10.31 ± 1.755 11.93 ± 1.710	P1 = 0.634 P2 = 0.845

P1 Pre-intervention comparison

P2 Post-intervention comparison

Data are mean (SD) or number

### Use and costs of resources

The average total medical costs per patient were significantly higher in the control group compared to the intervention group (Table 3). It is worth noting that there was no statistically significant difference in pharmacy costs between the two groups (*P* = 0.365), with the main disparity lying in rehabilitation medical expenses incurred by the control group during this period. This study found that non-medical costs were significantly higher in the intervention group compared to the control group (*P* < 0.001). This was mainly due to human cost of volunteers and administrative costs in the intervention group, whereas travel costs accounted for a very small proportion. In the intervention group, we employed a professional and systematically trained team of volunteers to provide support and assistance. These costs were necessary in the intervention group. In addition, the intervention group also needs to invest more management costs. In contrast, the control group spent less on these areas. There was no significant difference in time cost of patient and caregiver between the two groups (*P* = 0.097; Table 3).

**Table 3:** The cost comparison between the intervention group and the control group

Variable	Intervention group Mean Cost (95% CI) CNY	Control group Mean Cost (95% CI) CNY	*P* Value
**Medical costs**			
Direct medical costs	–	10,132 (8746.70–11,518.22)	–
Pharmacy costs	1001.83 (862.88–1140.79)	1112.76 (904.64–1320.88)	0.365
**Total direct costs**	1001.83 (862.88–1004.44)	11,245.22 (9795.93–12,694.51)	< 0.001
**Nonmedical Costs**			
Time cost of caregivers	1522.40 (1087.36–1957.44)	2760.00 (2144.39–3375.61)	0.097
Time cost of patients	2484.00 (1929.95–3038.05)	1370.16 (978.62–1761.70)	0.097
Travel costs	629.33 (303.94–954.72)	–	–
Human cost of volunteers	2912.00 (2611.15–3212.85)	130.13 (103.63–156.64)	< 0.001
Cost of training and materials	18.20 (15.66–20.74)	17.87 (15.20–20.54)	0.801
Administrative costs	1456.00 (1305.58–1606.42)	65.07 (51.81–78.32)	< 0.001
**Total Nonmedical costs**	4024.32 (3181.34–4867.30)	5629.95 (4546.18–6713.73)	0.032
**Total costs**	8889.76 (7801.97–9977.55)	15,675.81 (13,561.09–17,790.54)	< 0.001

### Cost-effectiveness analysis

With swallowing function, depressive symptoms, and quality of life as the effect indicators, Patients undergoing conventional rehabilitation incurred an additional cost of 3590 yuan per improvement in swallowing function score, 9979 yuan per improvement in depressive symptoms, and 1152 yuan per point increase in quality of life (Table 4).

**Table 4:** Cost-effectiveness results from trial data

Cost-effectiveness	ICER CNY
ICER, per Improved swallowing	−3590 (Dominant)
ICER, per Improved depression	−9979 (Dominant)
ICER, per Improved QOL	−1152 (Dominant)

## Discussion

The present study revealed that participation in group exercise sessions held at a community center significantly improved swallowing function, alleviated depressive symptoms, and enhanced overall quality of life among stroke patients with dysphagia. In terms of dysphagia, both groups demonstrated improvements in swallowing function. However, in addition to providing specific training for post-stroke dysphagia, group training also step-by-step feeding guidance based on the patient’s current function, as evidenced by the comparison of FOIS scores, which is particularly important. The evidence suggests [6] that while bolus adjustments ensure the safe swallowing of liquids and food, however, patients may not proactively assess the benefits.

It is worth noting that these exercises were conducted in a community-based group training program, it also demonstrated benefits in improving depressive symptoms. The study findings demonstrated a significant decrease in depression within the intervention group, whereas the control group experienced a progression or exacerbation of depressive symptoms. We also acknowledge that depressive symptoms are prevalent among patients with dysphagia, which may increase the risk of dysphagia complications or accelerate symptom progression, and these findings are more relevant to the results of this study. Additionally, dysphagia may contribute to the development of depression over time. This finding highlights the importance of promoting physical activity among individuals with dysphagia, as they are more likely to experience depressive symptoms compared without dysphagia [22]. Community-based group rehabilitation provides a novel therapeutic experience that strengthens interpersonal relationships and mobilizes psychosocial and emotional resources, which are essential for progressive adaptation and reintegration into society. Research has demonstrated that engaging in physical exercise and maintaining positive social connections can enhance emotional well-being and facilitate functional and psycho-emotional benefits after stroke [23]. Therefore, It’s not surprising that there was a stronger correlation between participating in more leisure activities and experiencing fewer depression symptoms [24].

Furthermore, satisfaction from life was significantly associated with functional outcomes. Yet, a higher correlation was found with participation [25]. And group environment fosters functional and lifelike forms of communication, which is also a beneficial factor for enhancing patients’ quality of life [26]. At the end of this study, the total score of quality of life in the intervention group was significantly changed, especially in the sub-domain, the changes in symptoms, frequency, communication, and sleep in the intervention group were higher than those in the control group. So our intervention was successful to some extent. Participants reported that group rehabilitation program served as their motivation to stick with the course, enabling them not only to foster friendships and nurture hope for the future. They formed a WeChat exchange group to share experiences, and expressed sadness at the activity’s conclusion while remaining hopeful for its continuation. In this sense, community-based group rehabilitation training is particularly suitable for patients with dysphagia, especially in improving mood and quality of life in this population. The implementation of this treatment approach may have resulted in the interconnection of outcomes, leading to enhanced consistency in training, which may have positively influenced the gains of the intervention group.

### Cost-benefit analysis of group rehabilitation training

This analysis suggests that the group rehabilitation program is cost-effective in comparison to usual treatment, generating net cost savings. In terms of cost composition, the control group had a higher proportion of medical institutions’ costs. To improve compliance and management effectiveness, more emphasis was placed on the human costs in the intervention group. It should be pointed out that the preliminary plan of group rehabilitation program required a significant amount of human and organizational work. But it ensured the efficient use of resources and was cost-effective. Dysphagia after stroke needs scientific management and long-term active treatment, taking into consideration the affordability of patients.. At present, there are few literatures on economic evaluation of dysphagia after stroke in community management. This study has made a beneficial exploration and attempt at economically evaluating a group rehabilitation program after stroke. The results show that this method is feasible. It can be considered as the most economical and effective management mode, which can realize the rational allocation of limited health resources.

The limitations of this study and suggestions for future studies are as follows. Firstly, The sample size of this study is small, which may have resulted in underpowered analysis. Second, it was impossible to blind participants or people delivering the intervention. Third, The study lacked longer follow-up, which would also enable us to determine whether the loss to follow-up observed in the control group will worsen and if the effect in the intervention be maintained, as well as for how long.

## Conclusions

In conclusion, Community-based group rehabilitation program is a viable and cost-effective way to help stroke patients improve swallowing function, mood and quality of life. However, this method has not become the standard treatment for clinical rehabilitation in China. Further studies are needed to assess the feasibility of conducting group rehabilitation program in a community setting (such as a community center) and to follow up on the long-term benefits of participating in group rehabilitation program.

## Data Availability

Data are available from the corresponding author upon reasonable request.

## Abbreviations

SLP: speech-language pathologist
WHO: World Health Organization
GBsEMGBF: Game-based surface electromyographic biofeedback training
SWAL-QOL: Swallowing-related Quality of Life
GDS-15: 15-item Geriatric Depression Scale
SSA: Standardized Swallowing Assessment
FOIS: Functional Oral Intake Scale
